# Necrological

**Published:** 1881-10

**Authors:** 


### NECROLOGICAL.

" Death is the crown of life:
Were death denied, poor man would live in vain."

Dr. John E. Crowe, Prof. of Obstetrics in the University of Louisville, died suddenly on September 27th, in the office of his friend Dr. M. F. Coomes, of acute laryngitis. Dr. Crowe was 53 years old, and had occupied the chair of obstetrics in the Louisville University since 1868.

Prof. Otto Spiegelberg, of Breslau, the distinguished German obstetrician and gynecologist died in Breslau of contracted kidney on August 9. He was a writer of great force and originality.

Prof. Wm. Warren Greene, of Portland, Me., died at sea on his way home from International Medical Congress. He wrote a series of graphic letters on the work of the congress to the *Boston Medical and Surgical Journal.*

M. E. Littré, the great French lexicographer, positivist, and medical author, recently died at an advanced age. In an account of his work published some years ago in an interesting brochure entitled " Etudes et Glanures " he says: "Twenty-seven years in all I worked at the dictionary; ten years at the translation of Hippocrates; four years at the Edition of Pliny; three years on the revising of Nysten's Dictionary." He worked sixteen hours a day, with only a month's vacation a year.

Dr. Bryk, Professor of Surgery at the University of Cracow, Poland, died lately, in his sixty-second year.

